# Classic Visual Search Effects in an Additional Singleton Task: An Open Dataset

**DOI:** 10.5334/joc.182

**Published:** 2021-07-28

**Authors:** Kirsten C. S. Adam, Titiksha Patel, Nicole Rangan, John T. Serences

**Affiliations:** 1 Department of Psychology, University of California San Diego, US; 2 Neurosciences Graduate Program, University of California San Diego, US; 3 Institute for Neural Computation, University of California San Diego, US

**Keywords:** distractor suppression, stimulus history, attentional capture, additional singleton task

## Abstract

Visual search refers to our ability to find what we are looking for among many competing visual inputs. Here, we report the availability of a rich dataset that replicates key visual search effects and shows that these effects are robust to several changes to the experimental design. Experiment 1 replicates classic findings from an additional singleton visual search task. First, participants are captured by a salient but irrelevant color singleton, as indexed by slower response times when a color singleton distractor is present versus absent. Second, attentional capture by a color singleton is reduced when the visual search array contains heterogeneous shapes rather than homogenous shapes. Finally, attentional capture by a color singleton is reduced when the display colors are repeated rather than switched unpredictably from trial to trial. Experiment 2 demonstrates that these classic visual search effects are robust to small procedural changes such as task timing (i.e., a 2–8 second rather than ~1 second inter-trial interval). Experiment 3 demonstrates that these classic effects are likewise robust to changes to the distractor frequency (75% rather than 50%) and to fully blocking versus interleaving blocks of two task conditions. All told, this dataset includes 8 sub-experiments, 190 participants and >210,000 trials, and it will serve as a useful resource for power analyses and exploratory analyses of visual search behaviors.

## Introduction

Visual search refers to our ability to find what we are looking for in a cluttered visual world. For example, when searching for the remote, you need to look for items that match your goals (e.g., “black, square”) while ignoring salient distractors (e.g., bright flashes from your phone’s push notifications). In the lab, we use highly simplified search displays to measure the influence of salient distractors on visual search performance. In a typical laboratory task known as the “additional singleton task” (Theeuwes, 1991, 1992), participants search for a shape singleton target among non-target items (e.g., a green diamond among green circles). On some trials, one of the non-target items is a salient but irrelevant color singleton distractor (e.g., a red circle). Attentional capture in this task is quantified as the response time cost when the color singleton distractor is present versus absent (“distractor present” versus “distractor absent”).

Here, we employ 4 variants of an additional-singleton task in order to replicate classic visual search effects. In all variants, participants search for a diamond and report the orientation of the line inside (horizontal or vertical), and attentional capture is quantified as the response time difference for singleton distractor present versus absent trials. In the first task variant, non-target items are homogenous shapes (i.e., participants search for a diamond target among circles), and the colors swap randomly from trial to trial (i.e., participants are equally likely to encounter: (1) all green items, (2) green items with a red singleton distractor, (3) all red items or (4) red items with a green singleton distractor). In the second variant, non-target items are heterogeneous shapes (i.e., participants search for a diamond target among circles, triangles, pentagons, and hexagons), and the colors swap randomly from trial to trial. Based on prior work, we expect that the color singleton distractor will capture attention more effectively when non-target shapes are homogeneous, because participants may adopt a “singleton-detection” strategy in which they direct attention toward the most salient display features, rather than a “feature search” strategy in which they strategically search for items that match the search template (e.g., Bacon & Egeth, 1994; Pashler, 1988).^1^ The final 2 variants are the same, except that colors are held constant in the entire block (e.g., the majority of items are always red, the singleton distractor is always green). Based on prior work, we expect that the color singleton distractor will no longer capture attention when colors are repeated over many trials (e.g., Vatterott & Vecera, 2012), but for ongoing debate see (Stilwell & Gaspelin, in press; Wang & Theeuwes, 2020).

Across three experiments, we replicate classic findings from the visual search literature, including (1) attentional capture by an irrelevant color singleton (Pashler, 1988; Theeuwes, 1991, 1992) (2) reduced attentional capture by the color singleton for heterogeneous versus homogeneous non-target shapes (Bacon & Egeth, 1994; Lamy et al., 2006; Leber & Egeth, 2006) and (3) reduced attentional capture by the color singleton when colors are repeated over time (Gaspelin et al., 2017; Vatterott & Vecera, 2012; Won & Geng, 2020). Originally, these experiments were used to ensure that small changes to typical procedures would not perturb expected behavioral effects (as some changes were needed to make this task amenable to fMRI: Adam & Serences, 2021). We found that the core visual search effects were highly consistent across small changes to the task procedures (including: proportion of distractor-present trials, length of the inter-trial interval, blocking or interleaving of task conditions, jittered versus fixed item locations). Across experiments, we collected a large number of participants and trials, so we anticipate that this dataset will be useful for further exploratory analyses (e.g., modeling) and for generating power estimates for future studies.

## Methods

### Participants

Participants were recruited from the University of California San Diego and surrounding community. Participants provided written informed consent, and the procedures were approved by the local Institutional Review Board. Participants were at least 18 years of age and had normal or corrected-to-normal visual acuity and normal color vision. The number of participants per experiment, the number of trials per participant, and the average self-reported age and gender of participants are shown in ***Table 1***. Across all experiments, a total of 3 participants were excluded for low task accuracy (<55%), 2 datafiles were excluded for being incomplete (e.g., the participant pressed the “escape” key after a few trials and was restarted as the next participant number) and 9 participants were excluded from Experiment 3B because we made changes to the stimulus size, and we wanted the full sample to perfectly match the stimulus parameters being used for a later study. Raw data for excluded subjects are available in the OSF repository. The main RT results from Experiment 3B are also reported in Adam & Serences (2021), but no other experiments have been previously reported.

**Table 1:** **Sample size, average age, and gender for each study**. “Included N” indicates the final number of subjects analyzed in each experiment; brackets indicate the number of additional excluded subjects not included in the analysis. Average age is shown in the age column; brackets indicate standard deviation. Gender: F = female, M = male, O = non-binary or other. The final row shows the total counts for N, trials, and gender, and the overall average for age. Note, raw data for excluded subjects are still available in the OSF repository.


EXPERIMENT	INCLUDED N [EXCLUDED N]	TRIALS/PARTICIPANT	AGE [SD]	GENDER

1a	24 [0]	1600	20.3 [1.8]	F – 18, M – 6, O – 0

1b	24 [2]	1600	20.7 [2.1]	F – 17, M – 7, O – 0

1c	24 [0]	1600	20.3 [1.7]	F – 16, M – 7, O – 1

1d	24 [2]	1600	19.9 [1.9]	F – 16, M – 7, O – 1

2a	24 [0]	640	20.3 [2.2]	F – 14, M – 10, O – 0

2b	22 [0]	640	20.0 [1.3]	F – 16, M – 6, O – 0

3a	24 [1]	576	21.5 [3.0]	F – 16, M – 8, O – 0

3b	24 [9]	576	19.8 [1.5]	F – 21, M – 3, O – 0

**OVERALL**	**190 [14]**	**210,688**	**20.3 [2.0]**	**F – 134, M – 54, O – 2**


### Stimuli

Stimuli were shown on a linearized Cathode Ray Tube (CRT) monitor (39 × 29.5 cm) from a viewing distance of ~52 cm. Stimuli were generated using a Linux computer (Ubuntu 16.04) running MATLAB 2016b (MathWorks, Natick, MA) with the Psychtoolbox extension (Kleiner et al., 2007; Pelli, 1997). Data were analyzed using MATLAB 2018a, Python (3.8.5, Python Software Foundation, *https://www.python.org*), and JASP 0.13.1 (JASP Team, 2020). Participants were seated in a dim room with their head resting on a chinrest.

In all experiments, stimuli were presented on a black background. Participants fixated a small white dot (.2°) at the center of the display, and the fixation dot remained visible throughout the entire experiment. Each item in the search array had a radius of 2.2° and appeared on an imaginary circle with radius 6.9° centered around fixation. Search items were equidistantly spaced around the imaginary circle and could be red (RGB = 255,0,0) or green (RGB = 0,255,0). On distractor absent trials, all items are the same color (green or red). On distractor present trials, all items are the same color except for a singleton distractor (i.e., all green with 1 red distractor or all red with 1 green distractor). We will use the term “majority green” or “majority red” to refer to the main display color. A small white line (.08° × .81°) appeared inside each search array item; each item was independently and randomly assigned one of two orientations (horizontal or vertical). These size values were the same for all experiment except Experiment 3B, which had the following small changes: imaginary circle radius = 7.0°, target line = .08° × .94°, search item radius = 2.4°.

### Task Procedures

#### Conditions

**Figure 1:**
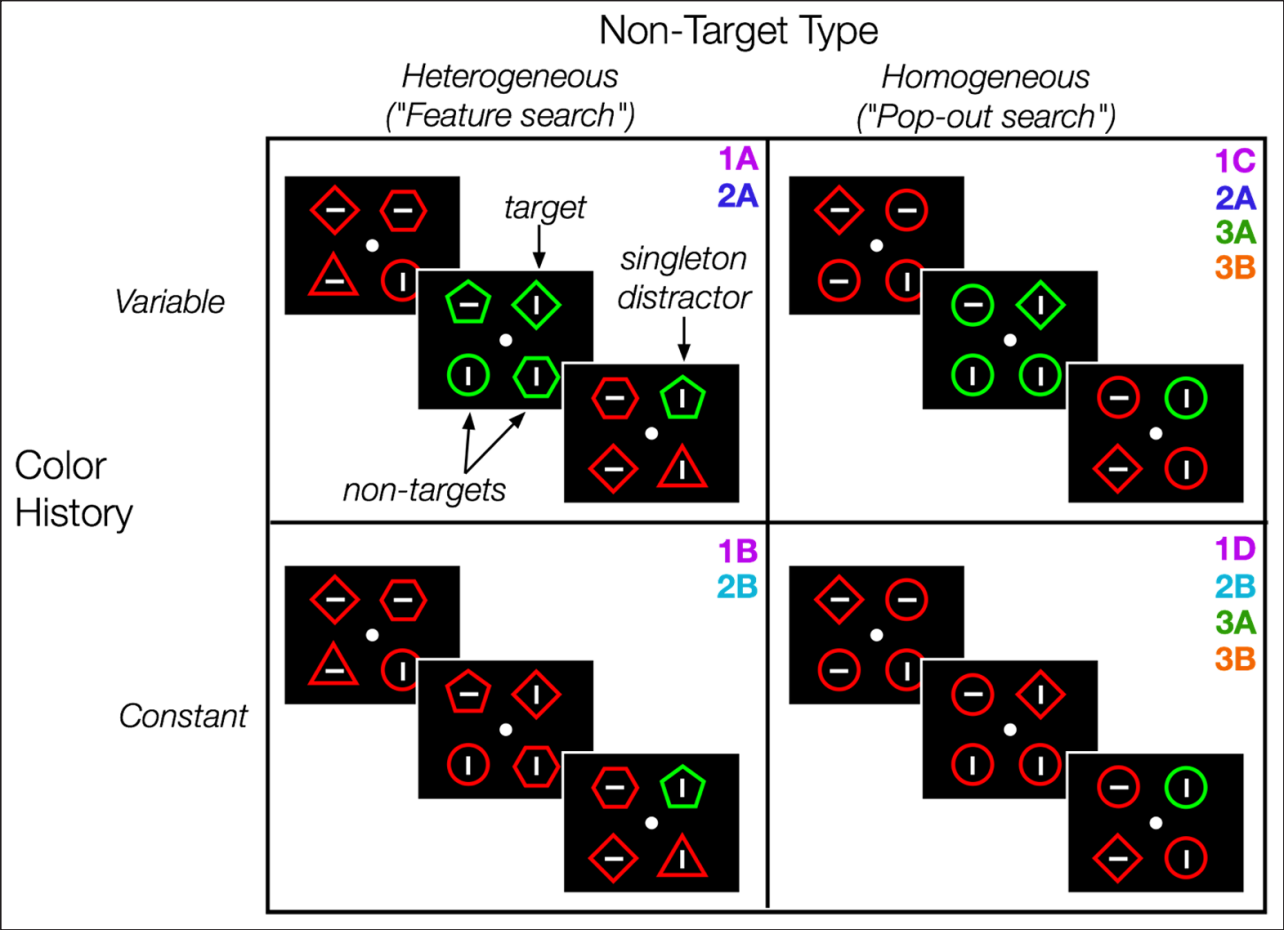
**Schematic of the task conditions**. On each trial of the task, participants searched for the target (diamond shape) and reported the orientation of the line inside (horizontal or vertical). After a blank inter-trial interval, the next search display appeared. Non-target shapes could be *heterogeneous* (assorted shapes) or *homogeneous* (all circles). The display colors could vary randomly from trial to trial (*color variable*) or stay constant within the entire block (*color constant*). The four conditions in this figure were collected across-subjects in Experiments 1a–1d. The conditions in the top row (Exp 2A) and bottom row (Exp 2B) were collected within-subjects in Experiment 2. The conditions in the right column were collected within-subjects in Experiment 3A and 3B.

The possible task conditions are depicted in ***Figure 1***, and which conditions occurred in each experiment are labeled in ***Figure 1*** and ***Table 2***. Participants performed these conditions in separate blocks. On each trial, the participants saw a display with multiple items, and their goal was to find the target (diamond shape) as quickly as possible and report via keypress whether the line inside the target was horizontal (“z” key) or vertical (“/” key). In the “heterogeneous” condition, the non-target shapes varied (shape set: circle, triangle, pentagon, hexagon). Non-target shapes were drawn from the shape set without replacement (set sizes 3–5) or without replacement from a doubled list of the shape set (set size 6), such that each non-target shape could be repeated no more than one time. In the “homogeneous” condition, the non-target shapes were all circles. We also varied trial history by manipulating whether the colors were repeated or switched from trial to trial. In the “color variable” condition, the colors switched randomly from trial to trial (50% switch probability; majority green or majority red). In the “color constant” condition, the colors were held constant within the entire block of trials (e.g., always green with a red distractor or vice versa).

**Table 2:** **Overview of experiments**. This table provides an overview of the differences between the experiments. Set Sizes refers to the number of items in the search array (3–6 intermixed, or only set size 4). Colors refers to whether the colors of the target and distractor were fixed across all trials (“color constant”) or randomly swapped from trial to trial (“color variable”). In experiments where both color conditions were included, the column Color Condition Balancing indicates whether the condition switched every other block of trials (“interleaved”) or if the conditions were grouped into the first and second halves of the experiment (“grouped”). The column Non-Target Type indicates whether the non-target shapes were heterogeneous (mixture of circles, triangles, and pentagons) or homogeneous (all circles). The column Timing indicates whether the study used typical behavioral timing (~1 second between trials) or longer timing (2–8 seconds between trials). Finally, the Distractor Present column indicates the proportion of trials where a singleton color distractor was present (50% or 75%). Between-subjects factors of interest are bolded for each experiment (e.g., the color and non-target shapes varied across sub-experiments 1A–D).


EXPERIMENT	SET SIZES	COLOR HISTORY	COLOR CONDITION BALANCING	NON-TARGET TYPE	TIMING	DISTRACTOR PRESENT

1a	3,4,5,6	**Variable**	–	**Heterogeneous**	Short	50%

1b	3,4,5,6	**Constant**	–	**Heterogeneous**	Short	50%

1c	3,4,5,6	**Variable**	–	**Homogeneous**	Short	50%

1d	3,4,5,6	**Constant**	–	**Homogeneous**	Short	50%

2a	4	**Variable**	–	Heterogeneous & Homogeneous	Long	50%

2b	4	**Constant**	–	Heterogeneous & Homogeneous	Long	50%

3a	4	Variable & Constant	**Interleaved**	Homogeneous	Long	75%

3b	4	Variable & Constant	**Grouped**	Homogeneous	Long	75%


#### Experiment 1

Each search array could contain between 3 and 6 items. These set size conditions were equally likely to occur, and they were randomly intermixed within each block. The positions of the search array were slightly jittered (i.e., the items were always spaced equidistantly from one another, but the rotation of this array was jittered uniformly around all possible angles). A color singleton distractor was present on 50% of trials. The search array disappeared as soon as the participant made their response. If the participant did not respond within 2 seconds, then the search array disappeared, and a non-response was recorded. The inter-trial interval lasted between 0.6 and 1.1 seconds (finely jittered, uniform distribution), and the next search array was presented immediately after the inter-trial interval elapsed.

#### Experiment 2

Each search array contained 4 items, and the positions were fixed such that the 4 items appeared at 45°, 135°, 225° and 315° on the imaginary circle around fixation. A color singleton distractor was present on 50% of trials. The search array disappeared as soon as the participant made their response. If the participant did not respond within 2 seconds, then the search array disappeared, and a non-response was recorded. The inter-trial interval lasted either 2, 3, 5, or 8 seconds (equal numbers of trials per ITI within each block in the “heterogeneous non-targets” condition in Experiment 2a and all conditions for Experiment 2b; due to a programming error, the inter-trial-interval (ITI) was held constant at 2 seconds for the “homogeneous non-targets” condition in Experiment 2a. Note, this programming error only affected the distribution of ITI values and did not affect any other part of the task. Specifically, in affected conditions, rather than choosing a value from a list of all 4 ITI values [2, 3, 5, 8], only the first value from the list [2] was used).

#### Experiment 3

Each search array contained 4 items, and the positions were fixed such that the 4 items appeared at 45°, 135°, 225° and 315° on the imaginary circle around fixation. A singleton distractor was present on 75% of trials, the search array was always shown for exactly 2 seconds (regardless of response), and task conditions were grouped or interleaved. The inter-trial interval lasted either 2, 3, 5, or 8 seconds (*Experiment 3A*: equal numbers of trials per ITI within each block for all conditions for all but the first 2 subjects. *Experiment 3B (and first 2 subjects of Experiment 3A)*: equal numbers of trials per ITI in the “color constant” condition and held constant at 2 seconds for the “color variable” condition). In Experiment 3A, the “color constant” and “color variable” conditions were interleaved within each subject (Block 1 = color constant (majority green), Block 2 = color variable, Block 3 = color constant (majority red), Block 4 = color variable, etc.). In Experiment 3B, blocks of different conditions were grouped together (e.g., Blocks 1–6 “color variable”, Blocks 7–9 “color constant” (majority red) and Blocks 10–12 “color constant” (majority green). The interleaved blocks were presented in a fixed order for all participants; the order of the grouped blocks was counterbalanced across participants (Table S2).

## Results

### Heterogeneity of non-target items has a large effect on overall search efficiency

**Figure 2:**
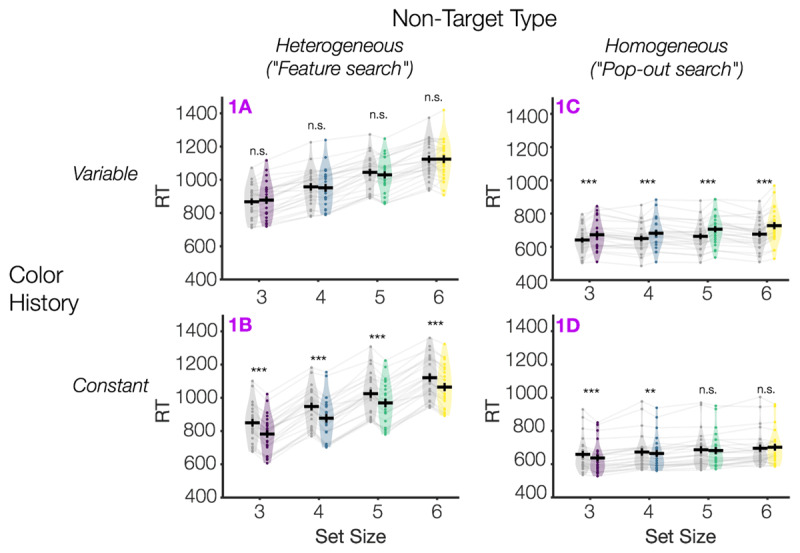
**Response times as a function of condition in Experiment 1**. We observed expected signatures of a singleton-detection strategy (homogeneous non-target shapes) versus feature search strategy (heterogeneous non-target shapes), and both singleton-detection and feature search were modulated by stimulus history (color constant vs. variable). Gray violins indicate “distractor absent” trials; colored violins indicate “distractor present” trials. Individual participants are shown as dots and transparent gray lines. Each subplot shows the response time data from a sub-experiment (**1A–1D**). Asterisks indicate uncorrected post-hoc comparisons between each adjacent pair of violins (distractor present vs. absent), *n.s. p* ≥ .05, ** *p* < .01, *** *p* <.001.

As expected, in Experiment 1 we replicated typical findings that the heterogeneity of non-target shapes has a large effect on search efficiency (***Figure 2***). We performed a mixed repeated measures ANOVA on response times with the between-subjects factors Color History (constant or variable) and Non-Target Type (heterogeneous vs. homogeneous) and the within-subjects factors Set Size and Distractor Presence. We found that Non-Target Type had a large effect on search times, *F*(1,92) = 202.99, *p* < .001, η^2^_p_ = .69, such that the effect of set size was much larger in the heterogeneous than homogeneous condition (Non-Target Type × Set Size, *F*(2.11,194.51)^2^ = 580.9, *p* < .001, η^2^_p_ = .86). This is consistent with prior findings that participants search relatively more serially when in “feature-search mode” and more in parallel when in “singleton detection mode”. However, search in the singleton-detection condition (homogeneous) was not perfectly parallel in our experiments – As also observed in Bacon & Egeth (1994), we found a slight but significant slowing of RT with set size in both Experiment 1C and 1D (*p* < .001; Table S1).

### Stimulus history has a large effect on attentional capture by salient singleton distractors

We likewise replicated expected findings that that Color History strongly modulates attentional capture by the salient singleton distractor. When the display colors switched unpredictably from trial to trial, participants were significantly slower when a singleton distractor was present versus absent. When the display colors repeated, capture by the distractor was reduced. This pattern of larger capture in the color variable (Exp 1A and 1C) versus constant condition (Exp 1B and 1D) was supported by a significant interaction of Color History × Distractor Presence in the main ANOVA, *F*(1,92) = 198.6, *p* < .001, η^2^_p_ = .68. In fact, the participants were so effective at suppressing the distractor in the color constant condition that we actually found distractor presence *benefits* whereby participants were faster when a singleton distractor was present than when it was absent. This was evident both in Experiment 1B and 1D. When participants were in feature-search mode (Exp. 1B), participants were much faster whenever a distractor was present, *F*(1,23) = 379.9, *p* < .001, η^2^_p_ = .94, and this distractor benefit did not interact with set size, *F*(3,69) = 1.98, *p* = .13, η^2^_p_ = .08. When participants were in singleton-detection mode (Exp. 1D), there likewise a main effect of distractor presence, *F*(1,23) = 6.14, *p* = .02, η^2^_p_ = .21, and this effect interacted with set size, such that participants were particularly fast on distractor present trials for low set sizes, *F*(3,69) = 10.6, *p* < .001, η^2^_p_ = .32. Task accuracy for all experiments is shown in Figures S1–S2, and we found no evidence of a speed-accuracy trade-off explaining either set-size or distractor presence effects.

### Expected search effects are robust to changes to task timing and condition balancing

**Figure 3:**
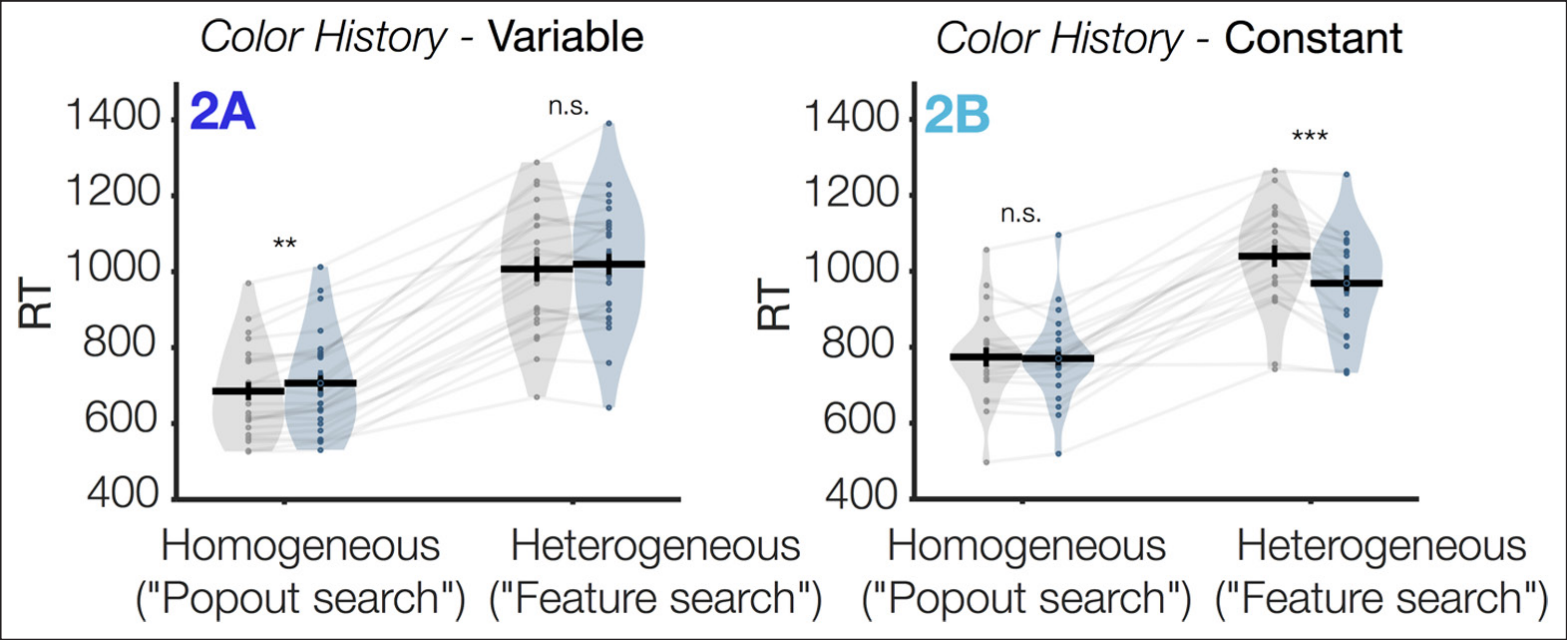
**Response times as a function of condition in Experiment 2**. Key visual search effects were preserved when a longer inter-trial interval was used (2–8 sec). Each subplot shows the response time data from a sub-experiment In Experiment 2, all displays were set size 4. Gray violins indicate “distractor absent” trials; colored violins indicate “distractor present” trials. Individual participants are shown as dots and transparent gray lines. Asterisks indicate uncorrected post-hoc comparisons between each adjacent set of bars, *n.s. p* ≥ .05, ** *p* < .01, *** *p* < .001.

In Experiment 2, we found that the typical visual search effects observed in Experiment 1 were robust to small changes to the task design (e.g., longer inter-trial interview; fixed rather than jittered item locations). We ran a mixed repeated measures ANOVA with the between-subjects factors Color History and the within-subjects factor Non-Target Type. Participants were again overall slower when non-target shapes were heterogeneous than homogeneous, as indicated by a main effect of Non-Target Type, *F*(1,44) = 424.1, *p* < .001, η^2^_p_ = .91, ***Figure 3***. Whether or not participants were captured by the color singleton distractor varied according to the particular condition, as indicated by a significant 3-way interaction of Non-Target Type, Distractor Presence and Color History, *F*(1,44) = 12.1, *p* = .001, η^2^_p_ = .22 (all 2-way interactions also significant). Specifically, when the display colors changed unpredictably from trial to trial (*Color Variable*, ***Figure 3***, Exp 2A), participants were captured by the singleton distractor when non-targets were homogeneous (“pop-out search”, *p* < .01), but were not captured when non-targets were heterogeneous (“feature search”, *p* > .05).^3^ When colors were held constant from trial to trial (*Color Constant*, ***Figure 3***, Exp 2B), participants were no longer captured by the distractor (Exp 2A, *p* > .05) or even were *faster* when the color singleton distractor was present (Exp 2B, *p* < .001).

**Figure 4:**
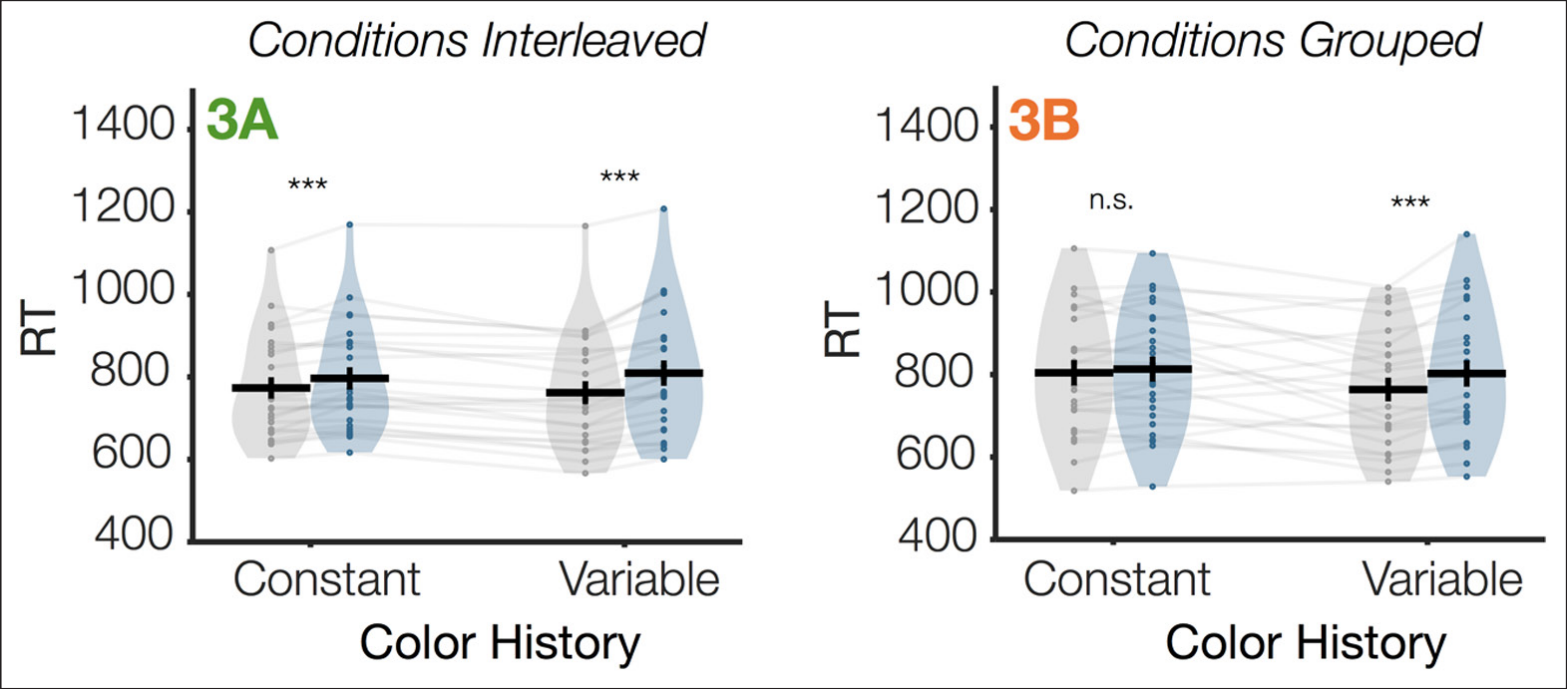
**Response times as a function of condition in Experiment 3**. When participants are in singleton-detection mode, attentional capture by a salient distractor is attenuated when colors are repeated (Color Constant) compared to when colors randomly vary (Color Variable). This general pattern did not differ as a function of interleaving (**3A**) versus grouping (**3B**) blocks of the two stimulus history conditions. In Experiment 3, all displays were set size 4 and non-target items were always homogeneous (singleton-detection mode). Gray violins indicate “distractor absent” trials; colored violins indicate “distractor present” trials. Individual participants are shown as dots and transparent gray lines. Asterisks indicate uncorrected post-hoc comparisons between each adjacent set of bars, *n.s. p* ≥ .05, **p < .01, ***p < .001.

Finally, in Experiment 3, we found that expected visual search effects were robust to other small changes to the task design (e.g., 75% rather than 50% distractor present trials; different condition counterbalancing schemes). We ran a mixed repeated measures ANOVA with the between-subjects factors Condition Balancing and the within-subjects factor Color History, and we found expected behavioral effects of Color History on search behavior. Specifically, we found that participants were significantly less captured by the distractor in the color constant condition compared to the color variable condition, as indicated by a significant Color History × Distractor Presence interaction, *F*(1,46) = 21.2, *p* < .001, η^2^_p_ = .32. This general pattern was not meaningfully affected by Condition Balancing, as indicated by no significant 3-way interaction of Color History × Distractor Presence × Condition Balancing (*p* = .64). However, post-hoc analyses indicate that distractor suppression was numerically more effective in the grouped experiment than in the interleaved experiment (***Figure 4***). Specifically, whereas capture by the distractor was non-significant in the Color Constant condition for the grouped experiment (Exp 3B), capture in the Color Constant condition was attenuated but still overall significant in the interleaved condition (Exp 3A).

## Discussion

Here, we report the availability of a large visual search dataset (8 sub-experiments, 190 subjects and >210,000 trials) that replicates several classic findings and shows that these classic findings are robust to small procedural changes. For example, these experiments show greater search efficiency for homogeneous versus heterogeneous non-target shapes (Bacon & Egeth, 1994; Duncan & Humphreys, 1989; and/or target/non-target similarity, see Mihali & Ma, 2020; Treisman & Gelade, 1980) and reduced attentional capture when item colors are repeated over time (Gaspelin et al., 2015, 2017; Geng et al., 2019; Turatto & Pascucci, 2016; van Moorselaar & Slagter, 2019; Vatterott & Vecera, 2012; Won & Geng, 2020). Indeed, in some cases, we even found distractor presence *benefits* whereby participants found the target faster when a salient singleton distractor was present. Because of the fairly large number of trials per participant that we collected (e.g., 1600 in Experiment 1) we anticipate this dataset will be useful for modeling the effects of stimulus history and non-target homogeneity (Calder-Travis & Ma, 2020; Mihali & Ma, 2020; Rosenholtz, 2001; Tseng et al., 2014) as well as for sub-sampling analyses to estimate the effect of trial counts on expected power for new within- and between-subjects task variants (Adam et al., 2020; Baker et al., 2020; Ngiam et al., 2021; Xu et al., 2017).

In addition to the primary response time metric (reported here), we also recorded other potential variables of interest on each trial. These variables include trial accuracy, whether a response was made, the color and position of each item, the inter-trial interval duration, the shape of each item, and the orientation of the line inside each item. Thus, we anticipate that this dataset will also be useful for other exploratory analyses. For example, in an additional analysis of Experiment 3b (Adam & Serences, 2021), we replicated the finding that capture shows a spatial gradient, whereby singleton distractors more strongly capture attention when they are near the target (Feldmann-Wüstefeld et al., 2021; Mounts, 2000).

In the online data repository (*https://osf.io/u7wvy/*), we have provided the raw data and analysis files in several formats that may be useful for pedagogical purposes. The original raw data, task code, and analysis scripts are all in MATLAB (*.m* and *.mat* files). In addition, we have provided general-use files (*.csv*) for flexible analysis of response times with most contemporary analysis programs. For example, we have provided an example of plotting and analysis of the data in either Python (via a Python notebook, *.ipynb*) or in the open-source software JASP (JASP Team, 2020). Together, we hope that this public data and code repository will provide a resource for learning to analyze visual search data, as well as a source of data for future exploratory analyses of visual search behaviors.

## Data Accessibility Statement

Data and code are available on the Open Science Framework at *https://osf.io/u7wvy/*

## Additional File

The additional file for this article can be found as follows:

10.5334/joc.182.s1 Supplemental Information. Supplementary figures and tables.

## Footnotes

Note, this work’s use of the term “feature search” differs from some other work (e.g., Treisman & Gelade, 1980, “feature search” vs. “conjunction search”).

Greenhouse-Geisser values are reported here and whenever the assumption of sphericity is violated (Mauchly’s test).

Note, for concision we often write ANOVA results as supporting an effect (capture) or no effect (no capture). Where no effect is found, this should be interpreted as “no evidence of an effect”, rather than evidence for the null.
