# Supplementary material for: Classic Visual Search Effects in an Additional Singleton Task: An Open Dataset
Source: J Cogn. 2021 Jul 28;4(1):34. doi: 10.5334/joc.182 (PMC8323537; doi:10.5334/joc.182)
Supplement: Supplemental Information. — Supplementary figures and tables. [file joc-4-1-182-s1.pdf]

Exp.	Condition Factor Label	Condition Factor	Distractor Presence	Condition x Distractor Presence
1a	Set Size	$F = 516.8$, **$p < .001$**, $\eta^2_p = .96$	$F = .33$, $p = .57$, $\eta^2_p = .01$	$F = 2.15$, p = .10, $\eta^2_p = .137$
1b	Set Size	$F = 651.1$, **$p < .001$**, $\eta^2_p = .97$	$F = 379.9$, **$p < .001$**, $\eta^2_p = .94$	$F = 1.98$, $p = .13$, $\eta^2_p = .08$
1c	Set Size	$F = 50.02$, **$p < .001$**, $\eta^2_p = .69$	$F = 83.95$, **$p < .001$**, $\eta^2_p = .79$	$F = 7.96$, **$p < .001$**, $\eta^2_p = .26$
1d	Set Size	$F = 68.8$, **$p < .001$**, $\eta^2_p = .75$	$F = 6.14$, **$p = .02$**, $\eta^2_p = .21$	$F = 10.6$, **$p < .001$**, $\eta^2_p = .32$
2a	Distractor Shape	$F = 313.99$, **$p < .001$**, $\eta^2_p = .93$	$F = 7.37$**, $p = .01$,** $\eta^2_p = .24$	$F = .52$, $p = .48$, $\eta^2_p = .02$
2b	Distractor Shape	$F = 136.3$, **$p < .001$**, $\eta^2_p = .87$	$F = 21.29$, **$p = <.001$**, $\eta^2_p = .50$	$F = 29.65$, p **$< .001$**, $\eta^2_p = .59$
3a	Colors	$F = .01$, $p = .91$ $\eta^2_p < .001$	$F = 69.85$, **$p < .001$**, $\eta^2_p = 75$	$F = 11.13$, **$p = .003$**, $\eta^2_p = .33$
3b	Colors	$F = 3.07$, $p = .09$, $\eta^2_p = .12$	$F = 44.07$, **$p < .001$**, $\eta^2_p = .66$	$F = 10.46$**, $p = .004$,** $\eta^2_p = .31$

Table S1. Summary of the repeated measures ANOVAs run within each sub-experiment. Every experiment includes the factor Distractor Presence (color singleton absent vs. present) and one other factor. The column Condition Factor Label describes the other factor that was manipulated in each study (Set Size: 3, 4, 5, or 6 display items; Distractor Shape: Homogenous versus heterogeneous shapes for non-target items; Colors: Color of target and distractor constant across the whole block or variable).

Exp.	Condition Counterbalancing Levels	Description
1a	1	There was only 1 condition (no counterbalancing)
1b	2	`p.counterbalance =1`: This subject only saw green targets (red singleton distractor)<br>`p.counterbalance =2`: This subject only saw red targets (green singleton distractor)
1c	1	There was only 1 condition (no counterbalancing)
1d	2	`p.counterbalance =1`: This subject only saw green targets (red singleton distractor)<br>`p.counterbalance =2`: This subject only saw red targets (green singleton distractor)
2a	2	`p.counterbalance = 1`: Pop-out search first half, feature search second half<br>`p.counterbalance = 2`: Feature search first half, pop-out search second half
2b	1	`p.counterbalance = 1`: Pop-out search first half, feature search second half. Target color always green.<br>`p.counterbalance = 2`: Pop-out search first half, feature search second half. Target color always red.<br>`p.counterbalance = 3`: Feature search first half, pop-out search second half. Target color always green.<br>`p.counterbalance = 4`: Feature search first half, pop-out search second half. Target color always red.
3a	4	There was only 1 condition order (no counterbalancing)
3b	2	`p.counterbalance = 1`: Color variable condition first half, color constant condition second half<br>`p.counterbalance = 2`: Color constant condition first half, color variable condition second half

Table S2. Summary of across-subject condition counterbalancing. Every experiment includes the factor Distractor Presence (color singleton absent vs. present) and one other factor. The column Condition Factor Label describes the other factor that was manipulated in each study (Set Size: 3, 4, 5, or 6 display items; Distractor Shape: Homogenous versus heterogeneous shapes for non-target items; Colors: Color of target and distractor constant across the whole block or variable).

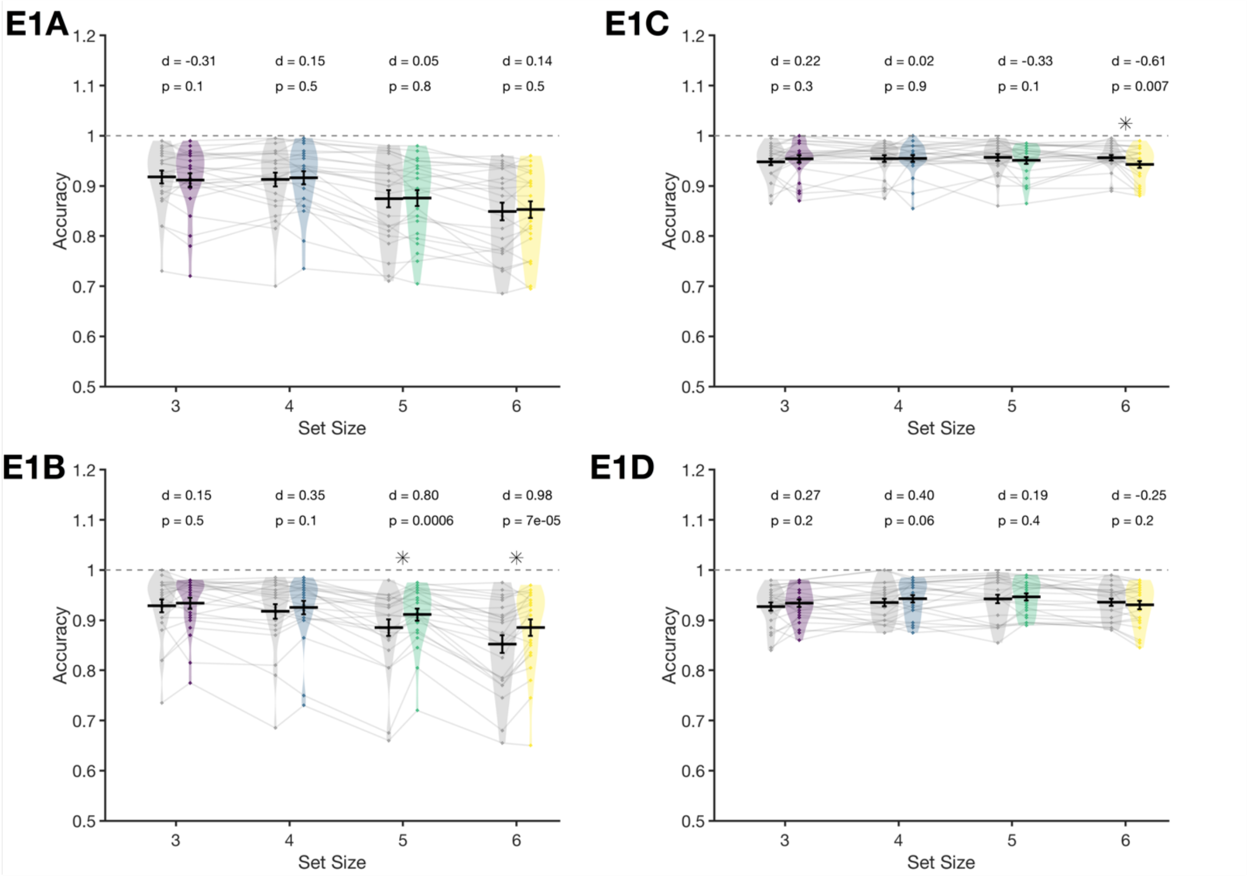

Figure S1. Response accuracy in Experiments 1A-1D.

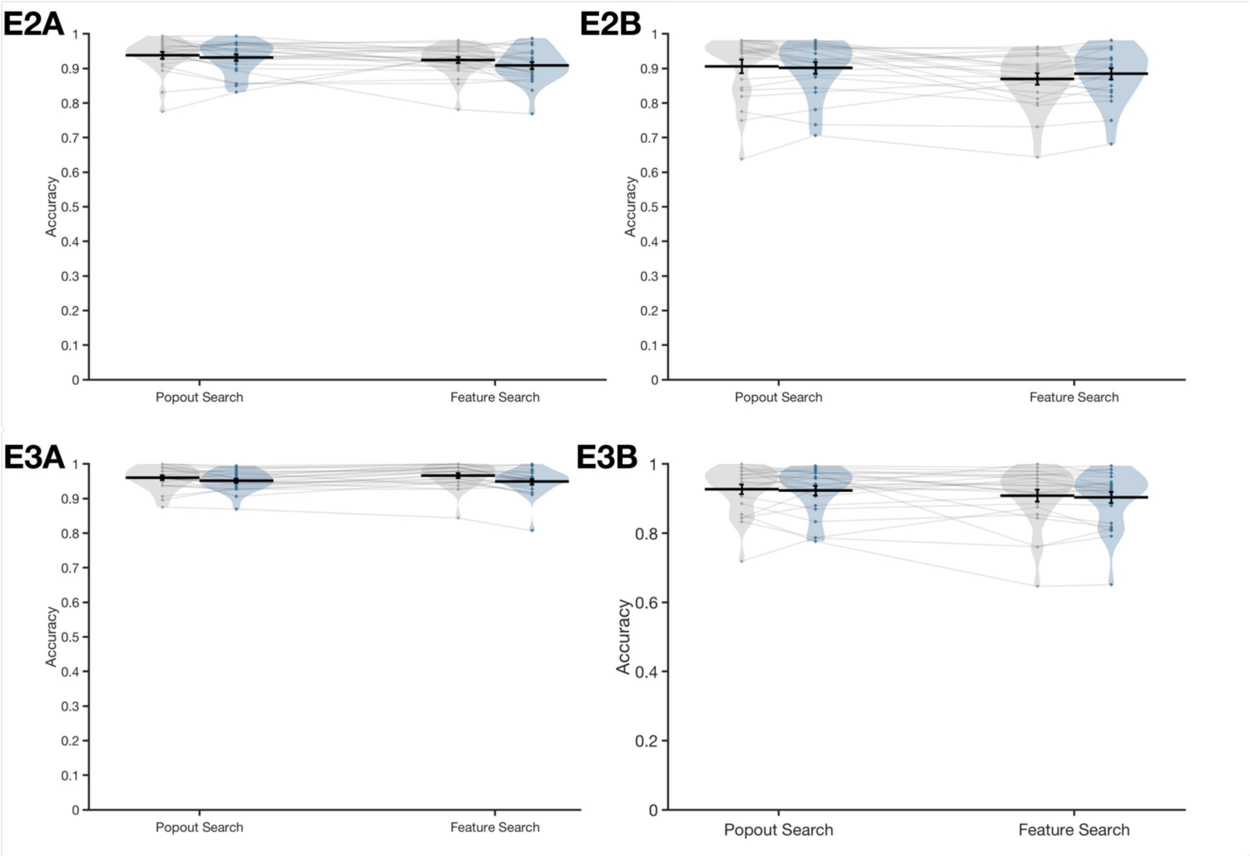

Figure S2. Response accuracy in Experiments 2 and 3.
